# Web-based need-supportive parenting program to promote physical activity in secondary school students: a randomized controlled pilot trial

**DOI:** 10.1186/s12889-023-16528-4

**Published:** 2023-08-25

**Authors:** Pille-Riin Meerits, Henri Tilga, Andre Koka

**Affiliations:** Institute of Sport Sciences and Physiotherapy, University of Tartu, Ujula 4, Tartu, 51008 Estonia

**Keywords:** Children, Adolescents, Self-determination theory, Intervention, Basic psychological needs, Autonomy, Competence, Relatedness, Motivation, Theory of planned behaviour

## Abstract

**Background:**

Current global trend of insufficient physical activity (PA) among children and adolescents highlights the necessity of finding effective ways to promote PA in childhood. Self-determination theory (SDT) has demonstrated efficacy as a conceptual framework for developing interventions aimed at promoting diverse health behaviours. Parents have potential to influence children’s health behaviours to a great extent, which could be enhanced from an online, self-paced training to gain knowledge on how to support children’s intrinsic motivation towards particular health behaviour. In this pilot study, we developed and tested an online SDT-informed need-supportive training for parents, enabling them to interact with their children in a way to support their intrinsic motivation towards leisure-time physical activity.

**Methods:**

Sixty eight students (M_age_ = 12.5 ± 0.72) and one parent for each child were randomly assigned to the 6-week intervention condition or control condition. Students completed psychological measures (i.e., perceptions of parents’ need-supportive behaviours, basic psychological need satisfaction and frustration, autonomous and controlled forms of motivation, as well as social cognition beliefs towards leisure-time PA) and self-reported PA pre-intervention, post-intervention, and one-month after the intervention. Repeated measures ANOVAs were conducted to test the effects of the intervention condition and time.

**Results:**

While a statistically significant intervention effect on children’s leisure-time PA was not found, students in the intervention group reported higher, albeit marginal, perceptions of intrinsic motivation (*F*(2, 84) = 3.095, *p* = 0.050) and lower perceptions of introjected regulation (*F*(2, 88) = 3.107, *p* = 0.050) and autonomy frustration (*F*(2, 84) = 2.987, *p* = 0.056) at follow-up. Contrary to expectations, children in the control group demonstrated higher perceptions of intention (*F*(2, 84) = 4.838, *p* = 0.010) and effort (*F*(2, 80) = 3.473, *p* = 0.036) towards leisure-time physical activity at follow-up. No significant changes were found in perceptions of need-supportive behaviour from parents, attitude, and perceived behavioural control.

**Conclusions:**

Our pilot study highlights the importance of parental training and the potential for SDT-informed interventions to support children's intrinsic motivation towards physical activity. Further research is needed to test the intervention in other domains and combine interventions in several domains to have the highest impact.

**Trial registration:**

This pilot study is part of preparation for the main study, prospectively registered in ISRCTN registry as ISRCTN78373974 (15.12.2022). The current stage of the main study is ‘recruiting’.

**Supplementary Information:**

The online version contains supplementary material available at 10.1186/s12889-023-16528-4.

## Background

Physical activity (PA) has many known benefits for children and adolescents [1, 2]. In addition to physical adaptive health outcomes such as reduced cardio-metabolic risk factors [3], the benefits can also be psychological such as reduced anxiety [4] and cognitive such as better academic performance [5]. On the other hand, physical inactivity has a detrimental effect on health, frequently leading to overweight that is difficult to lose later in life, and thus insufficient PA in childhood can result in a higher risk of non-communicable diseases such as cardiovascular disease as an adult [6]. Although WHO recommends that children and adolescents should get an average of 60 min of moderate-to-vigorous physical activity (MVPA) per day [7], the worldwide problem is that most children and adolescents to not meet the guideline levels of PA [8]. For example, data from 146 countries indicated that in 2016 more than 80% of adolescents were insufficiently active [8]. In Estonia, where the current pilot study was conducted, the proportion of children and adolescents meeting the WHO recommendation on PA has increased comparing 2018 and 2021 (28% to 43%), but even so less than half of the children get enough MVPA daily [9, 10]. Moreover, PA levels in adolescents decline with age [11, 12]. Specifically, Rubín and colleagues demonstrated that during the transition from childhood to adolescence sedentary time increases at the expense of PA [13].

PA in childhood predicts adult PA levels – being active as a child significantly increases the probability of being active as an adult [14]. As PA patterns tend to carry forward into adulthood, it is important to develop effective interventions to promote PA in children and adolescents. Researchers have tried to identify the determinants of PA that could be modified to result in higher activity levels.

One of the prominent motivational theories frequently used in developing health interventions is the self-determination theory (SDT) [15]. Deci and Ryan differentiate between several forms of motivation ranging from extrinsic to intrinsic. According to the SDT, a person’s intrinsic motivation is higher when his/her innate psychological needs for autonomy, competence, and relatedness are satisfied. The need for autonomy indicates that a person wants to feel s/he has a choice when it comes to what is happening in his/her life; the need for competence refers to one's desire to cope with difficulties and experience success; and the need for relatedness means that a person wants to be surrounded by caring companions, to be accepted by others. For optimal performance and wellbeing, social environment, including supportive behaviour from parents, peers, teachers etc., should enable satisfying all three basic psychological needs [16]. One of the main premise of SDT is that when a person’s psychological needs are fulfilled in an activity, development of intrinsic motivation towards this activity is facilitated [17]. This suggests that to promote PA in children and adolescents, interventions to support their basic psychological needs in the PA context could be used. Earlier intervention studies to support basic psychological needs have used various approaches and the obtained effects also vary [18]. Teixeira and colleagues [19] conducted a study using the Delphi method to identify techniques to support basic psychological needs. As a result, they classified 21 motivation and behaviour change techniques (MBCTs) that are used in SDT-informed interventions and described 7 techniques for satisfying each of the three psychological needs. Overall, research based on the SDT in the health domain has demonstrated that SDT-informed interventions positively affect health behaviour [18].

Understanding the determinants of PA in adolescents has been greatly advanced by the application of the trans-contextual model (TCM) [20, 21]. TCM is a multi-theory model, integrating components from SDT, the theory of planned behaviour (TPB) and the hierarchical model of intrinsic and extrinsic motivation [22]. SDT differentiates between autonomous and controlled types of motivation towards particular behaviour (e.g., PA) and TPB states that a person’s intention, strongly supported by autonomous motivation, is the most proximal predictor of PA behaviour. The hierarchical model describes how autonomous motivation in one context influences it in another context. The integration of these theories is based on the premise that they are complementary, with SDT and TPB explaining the reasons behind motivated behaviour and the hierarchical model being the connecting element between contexts [20].

Research based on TCM has revealed that adolescents who perceive greater autonomy support in physical education (PE) are more likely to engage in PA outside of school, due to increased autonomous motivation, social cognition beliefs (i.e., attitude, subjective norms, and perceived behavioural control), and intentions towards PA [20, 21]. Numerous intervention studies have been carried out in the school context, with PE teachers adopting need-supportive techniques [23, 24]. It has also been demonstrated that need satisfaction in PE classes and the amount of out-of-school MVPA are correlated, with the decrease of need satisfaction also decreasing PA levels [25].

The home setting has not been extensively studied so far. However, parents are also important influencers regarding children’s health behaviours. Parental support is significantly correlated to children’s PA levels [26, 27], so educating parents about need-supportive behaviours should help increase children’s PA.

“Active 1 + FUN” is an example of a family-based intervention program designed based on the tenets of SDT. The 6-month program was designed to help parents support their children’s basic psychological needs and increase children’s MVPA and co-activity with parents. The intervention was carried out using face-to-face approach and comprised of ten activity sessions. The program resulted in improving children’s fundamental moving skills, however changes in accelerometer-measured MVPA were not evident [28, 29]. The FRESH (Families Reporting Every Step to Health) study, an online family-based PA intervention program, demonstrated that children in the intervention group enjoyed participating in the study with their family members and reported more PA. Nevertheless, children’s accelerometer-measured MVPA was not significantly different between study groups after the 8-week intervention [30, 31]. Both “Active 1 + FUN” and FRESH studies had the limitation that target sample size was not reached, which might be one of the reasons for the lack of significant intervention effects [29, 32]. Furthermore, in the FRESH study randomization by county was used, which resulted in unbalanced study groups [31].

Behavioural interventions can harness digital technology to improve scalability and effectiveness [33]. Reeve and Cheon [34] have suggested that need-supportive interventions could be delivered in an online format, offering the possibility for more personalization and self-paced learning. Tilga and colleagues [35] have demonstrated that an entirely web-based intervention program for PE teachers was effective in enhancing students’ perceptions of autonomy-supportive teacher behaviours and students’ need satisfaction. The effects of the study were also evident at 15-month follow-up and in regard to intrinsic motivation even more pronounced [36]. Therefore, a web-based intervention may be an optimal and effective option to apply in future.

### The present study

In light of the empirical finding above, we developed an entirely web-based intervention program for parents, considering that self-paced online learning might be convenient for parents. The aim of the pilot study was to test the effectiveness of an online need-supportive study program to help parents interact with their children in a such way as to support their basic psychological needs (i.e., autonomy, competence, relatedness) and thereby support their intrinsic motivation towards out-of-school PA. The online study program for parents consisted of short educational videos to teach them effective communication techniques to use with their children. It also featured multiple choice tests about the videos and provided a discussion forum for exchanging experiences in implementing the techniques in real life situations. A more detailed description of the intervention program can be found in the methodology chapter in the Web-Based Need-Supportive Parenting Program subsection.

We expect that, at follow-up, children whose parents participate in the program demonstrate (1) significantly higher perceptions of parents’ need-supportive behaviours towards autonomy, competence, and relatedness; (2) higher perceptions of basic psychological need satisfaction; (3) lower perceptions of basic psychological need frustration; (4) higher perceptions of autonomous motivation; (5) lower perceptions of controlled forms of motivation; and (6) higher actual self-reported participation in out-of-school PA compared to the children whose parents were assigned to the control group.

We also expect increase in perceptions of the secondary outcome variables such as attitude, subjective norms, perceived behavioural control, intention, and effort towards PA at follow-up for children whose parents participate in the program relative to children whose parents are assigned to the control condition.

## Methods

### Participants

To estimate the required sample size to test the study hypotheses, power calculation was conducted using G*Power Version 3.1.9.7 [37]. The required sample size to achieve 75–80% power for detecting medium effects, at a significance level of *α* = 0.05, over three measuring points (baseline, post-intervention, one-month follow-up) was 76 to 86 participants for repeated measures ANOVA testing.

**Fig. 1:**
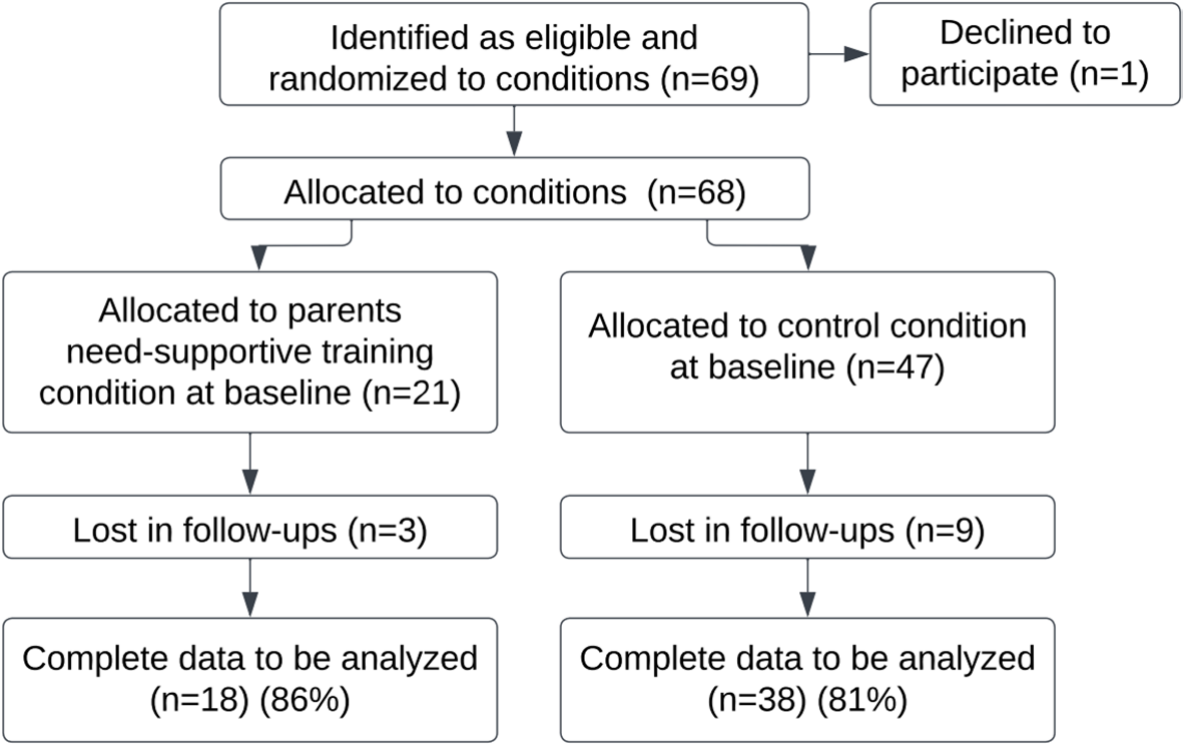
Participant flow diagram in the pilot study

We managed to recruit a total sample size of 68 children (28 boys, 40 girls) and one parent for each child who participated in the study. Students’ ages ranged from 11 to 15 years (*M* = 12.5 ± 0.72). The participant flow through the study is illustrated on Fig. 1.

Eligible participants were students without restrictions on their participation in PE classes and their parents. Recruitment was school based, and participants were cluster-randomized by school. Invitation letters were sent to randomly selected schools in Tartumaa County, Estonia. After confirmation, the researchers visited the classes 6–7 in consenting schools in person, introduced the study and distributed informed consent forms for students and parents. After a week, the filled-out consent forms were collected from schools, resulting in recruitment of student and parent pairs to participate in the study. The intervention was carried out during 2021/22 school year.

### Ethical considerations

The study was conducted in accordance with the Declaration of Helsinki and approved by the Research Ethics Committee of the University of Tartu (code: 327/T-4, 19.10.2020). We used the CONSORT checklist when writing our report [38].

Informed consent forms and study information were distributed by research team members, who also answered all questions that arose. Students were given information about the study, including the purpose, procedures, duration, potential benefits, and risks. Students were informed that participation is voluntary, and their anonymity would be guaranteed. To match the responses to questionnaires of one participant over three time-points, a personal code was generated for each student. The personal code was based on school name abbreviation, class number (“6” for sixth graders; “7” for seventh graders), participant’s initials, and gender (“B” for boys; “G” for girls). All questionnaires in three data collection occasions were marked with the personal code that was introduced to participants when distributing the first questionnaires. Data was transferred to electronic form without the assistant knowing the identity of the participants. The study did not harm the participants neither mentally nor physically, invasive research methods were not used.

### Experimental design

The study adopted a cluster-randomized controlled design with two study groups. Schools were randomly assigned to either intervention or control groups. Parents and their children were assigned to either intervention group or control group respective of the children’s school. Parents in the intervention group participated in a six-week web-based need-supportive intervention program. Students were blinded to allocation, they were not informed to which study group their school was assigned.

Measurements were taken in three time-points. In the pre-intervention data collection questionnaires containing baseline measures (i.e., demographics, psychological, and behavioural measures) were administered. The six-week training program was followed by post-intervention and one-month follow-up data collection occasions during which questionnaires with identical measures were administered again. In all data collection occasions, the questionnaires were distributed by researchers. The children were asked to fill in the questionnaires at home and return them in a closed envelope into the assigned box at school during the next week. Researchers collected the filled-in questionnaires from schools in 1–2 weeks after the distribution.

### Web-based need-supportive parenting program

A web-based format was chosen for the intervention as it is scalable, enables reaching larger audiences and offers the possibility of self-paced learning for the participants. The aim of the need-supportive parenting program was to educate parents about techniques directed at promoting children’s intrinsic motivation towards out-of-school PA. The parents in the intervention group had access to a learning platform (Moodle) for the duration of the study. An invitation link was sent to participating parents assigned to the experimental group to join the online course.

In the beginning of the program, the self-determination theory was introduced, emphasizing the role of basic psychological needs for autonomy, competence, and relatedness. Over the course of the intervention, parents were asked to watch three short video lectures each week. The course was self-paced, so that parents could watch the weekly videos whenever they had the time. Videos from the previous weeks were also accessible. On average, the duration of a single video was approximately 2 min. The videos covered a series of motivational and behavioural change techniques (MBCTs) that have been described by Teixeira and colleagues [19] as strategies to satisfy basic psychological needs. Described MBCTs are systematically divided into three groups, covering autonomy-support techniques (e.g., providing choice, using non-controlling informational language), competence-support techniques (e.g., offering constructive, clear, and relevant feedback, clarifying expectations), and relatedness-support techniques (e.g., showing unconditional regard, using empathic listening) [19]. We adapted the described techniques to the context of parent–child interaction and leisure-time PA. Each educational video introduced one behavioural technique to support one basic psychological need. The structure of the videos was as follows. First, it was explained which basic psychological need of the child the behavioural technique is designed to support in the context of leisure-time PA. Then the technique and the benefits associated with its application were explained. Finally, a sample video clip on the application of the technique in the form of parent–child interaction was presented. The content of the educational videos is described in more detail in Additional file 1.

Following designing and filming the preliminary educational videos, feedback was asked from a test group of parents (*N* = 12) about the understandability, possible effectiveness, and usability of the techniques. A 5-point Likert scale (1 = *strongly disagree* and 5 = *strongly agree*) was used for evaluation and the results revealed high scores on understandability (M = 4.86 ± 0.12), effectiveness (M = 4.41 ± 0.34), and usability (M = 4.29 ± 0.41). Since the feedback was positive, we proceeded with the pilot study using the developed video lectures.

Following each week’s educational videos, participants were asked to complete a short multiple-choice test to ensure they understood the techniques. In addition, a forum discussion was opened weekly, where participants could share their experiences trying to put the techniques into practice when interacting with their child daily. Overview of activities in Moodle revealed that parents were moderately engaged in the program. The percentages of parents taking the tests and participating in forum discussions were 64% and 22%, respectively.

### Outcome measures

For the outcome measures, students completed pen-and-paper questionnaires during pre-trial, post-trial and one-month follow-up data collection points. All measures were self-reported. Responses to items were collected using 7-point scales with endpoints meaning *strongly disagree* (1) and *strongly agree* (7), unless stated otherwise. The scoring of the measures was performed by summing the responses to the statements of each scale/subscale and dividing by the number of statements to obtain the scale/subscale mean.

#### Physical activity

The behavioural outcome measure was the children’s participation in out-of-school MVPA. The choice of MVPA as the behavioural measure was based on the many health benefits MVPA has for adolescents (e.g., better mental health, better cardio-respiratory fitness, and less fat gain) [39]. Students reported their PA behaviour by the short form of the International Physical Activity Questionnaire (IPAQ) [40], modified to explicitly refer to leisure-time PA. Responses to the IPAQ were converted to Metabolic Equivalent Task (MET) minutes per week according to the IPAQ scoring protocol. Total minutes of vigorous activity, moderate-intensity activity, and walking in the last seven days were multiplied respectively by 8.0, 4.0, and 3.3, resulting in MET scores for each of the activity levels [41]. Previous studies have demonstrated this measure to be reliable and valid [42] and it has been used in the Estonian context [43].

#### Perceived need support

To measure students’ perceptions of their parents’ need-supportive behaviours regarding out-of-school PA, the modified versions of the perceived autonomy support scale [44] and the need support scale [45] were used. Our used scale consisted of 13 items, four in autonomy support and competence support subscales, and five in relatedness support subscales. Example items are: “I feel that my parents provide me with choices, options, and opportunities about whether to do active sports and/or vigorous exercise in my free time” (perceived autonomy support); “I feel that my parents help me to improve in leisure-time PA” (perceived competence support); “My parents support me” (perceived relatedness support). Previous studies have demonstrated that the adapted perceived autonomy support scale is a reliable and valid measure to evaluate perceived autonomy support from parents [46] and it has been used in the Estonian context [46, 47]. The need support scale has also proven to be a reliable and valid measure [48] and it has been used in the Estonian context [48].

#### Psychological need satisfaction and frustration

Students’ perceptions of their autonomy, competence, and relatedness satisfaction and frustration in relation to leisure-time PA were measured by the Basic Psychological Need Satisfaction and Need Frustration Scale (BPNSNF) [49]. The BPNSNF scale has been adapted to the PE context by Haerens and colleagues [50] and we slightly modified the adapted version to refer specifically to leisure-time PA. The scale consists of four items for the satisfaction of each psychological need and four items for the frustration of each psychological need, resulting in a 24-item scale. All items were preceded by a common stem: “When I engage in PA during my free time…”. Example items are: “…I feel a sense of choice and freedom in the things I undertake” (autonomy satisfaction); “…most of the things I do feel like ‘‘I have to’’” (autonomy frustration); “…I feel that the people I care about also care about me” (relatedness satisfaction); “…I feel excluded from the group I want to belong to” (relatedness frustration); “…I feel confident that I can do things well” (competence satisfaction); “…I have serious doubts about whether I can do things well” (competence frustration). The BPNSNF scale has been demonstrated to be a reliable and valid measure and it has been used in the Estonian context [51].

#### Motivation towards PA

Autonomous and controlled forms of motivation towards out-of-school PA were measured using a modified version of the Perceived Locus of Causality Questionnaire [52]. The scale consisted of four subscales, each having two items. All items had a common stem: “I am physically active in my free time…”, and were followed by statements about intrinsic motivation (“…because I enjoy it”), identified regulation (“…because it is important to me to be physically active”), introjected regulation (“…because I feel bad about myself if I’m not physically active”), and external regulation (“…because others would not be satisfied with me if I’m not physically active”). Previous studies have demonstrated this measure to be reliable and valid [47, 53, 54] and it has been used in the Estonian context [47, 54].

#### Theory of planned behaviour constructs

Students’ attitude, subjective norms, perceived behavioural control (PBC), and intention for out-of-school PA were measured using the scales developed according to the recommended guidelines [55]. Attitude was measured by three 7-point scales with bipolar adjectives (unenjoyable/enjoyable, bad/good, useless/useful) following the stem “For me, participating in active sports and/or vigorous physical activities during my leisure time in the next 5 weeks is…”. Intention was measured by two items (e.g., “I intend to do active sports and/or vigorous physical activities during my leisure time in the next 5 weeks”). PBC was measured by two items (e.g., “How much control do you have over doing active sports and/or vigorous physical activities during your leisure time in the next 5 weeks?”), with responses collected using 7-point scales with end-points meaning *very little control* (1) and *full control* (7). Subjective norms were measured by two items (e.g., “Most people close to me expect me to do active sports and/or vigorous physical activities during my leisure time in the next 5 weeks”). Measures for the constructs of the theory of planned behaviour have been demonstrated to be reliable and valid [22, 46] and have been used in the Estonian context [47].

#### Perceived Effort

Students’ self-reported effort to do leisure-time PA was measured using the scale developed according to Hagger and Hamilton [56]. Effort was measured by two items (e.g., “During the last 5 weeks, how hard did you try to be physically active in your leisure-time?”). Responses were collected on a scale ranging from one (*did not try at all*) to seven (*tried extremely hard*). This measure has been shown to be reliable and valid [56, 57] and has been used in the Estonian context [57].

### Data analysis

The data analysis was conducted using JASP (Version 0.17.1; University of Amsterdam, Amsterdam, the Netherlands). Asymmetry values between -2 and + 2 and kurtosis values between -7 and + 7 were considered indicative of the data being normally distributed [58]. The range for skewness was from -1.809 to 1.271 and the range for kurtosis was from -0.993 to 5.711, so the collected data were in the normal distribution range. The reliability of used scales in the questionnaire was assessed using Cronbach’s alpha coefficient [59]. The Cronbach alpha values of the scales were mostly between 0.70 and 0.95, the full range being from 0.647 to 0.968, which suggests reliability of the questionnaire used. The extent of random missing values was on average 34% for different variables and all cases with data present for each variable were included in the analysis.

Randomization check to examine the baseline differences between study groups and attrition check to examine the differences between those who remain in the study and those who are lost to follow-up were conducted by using the independent samples *t*-test. In addition, chi-square tests were carried out to examine whether there were differences in gender across study groups as well as differences between children who remained in the study and who dropped out.

In the main analysis, a series of repeated-measures ANOVAs were used to test the effectiveness of the web-based need-supportive parenting program on the dependent variables (perceptions of parents’ need-supportive behaviours, basic psychological need satisfaction and frustration, autonomous and controlled forms of motivation, social cognition beliefs towards leisure-time PA and self-reported PA). Intervention condition (intervention vs control) was used as a between-subject factor and time (baseline vs post-intervention vs one-month follow-up) was used as a within-subject factor in each of the repeated measures ANOVAs. A post hoc analysis, independent measures *t*-test with Bonferroni adjustment was performed in case a significant effect appeared in any of the ANOVAs. By lowering the significance level for each individual test, the Bonferroni correction ensures that the overall probability of making a Type I error remains at an acceptable level [60]. By applying the Bonferroni adjustment, we aimed to maintain a more conservative approach to hypothesis testing in post hoc analysis, reducing the chances of false positives, and ensuring more reliable results. The significance level applied in this study was *p* < 0.05.

## Results

### Preliminary analysis

#### Randomization check

Table 1 shows characteristics of the participants at baseline. Results of the independent samples *t*-test revealed no significant differences in any of the study variables between the control and intervention groups at baseline (*t* = -1.655–1.384, *p* > 0.103). However, based on the chi-square test results, there was a significant difference in the proportion of male and female students across the intervention and control groups (*χ*^*2*^ = 8.150, *p* = 0.004).

**Table 1:** Comparisons of the baseline characteristics between the study groups

Variable	Intervention group (*n* = 21) *M* (SD)	Control group (*n* = 47) *M* (SD)	*t* or *χ*^*2*^ value	*p*
Age (in years)	12,71 (0,90)	12,40 (0,61)	*t* = -1.655	0.103
Gender (boy/girl)	14/7	14/33	*χ2* = 8.150	0.004
PAS from parents	6.05 (0.83)	5.92 (1.02)	*t* = -0.487	0.628
PCS from parents	5.91 (0.79)	5.80 (1.22)	*t* = -0.364	0.717
PRS from parents	6.39 (0.69)	6.11 (1.08)	*t* = -1.082	0.284
Intrinsic motivation	5.43 (1.61)	5.42 (1.44)	*t* = -0.030	0.976
Identified regulation	5.81 (1.43)	5.64 (1.44)	*t* = -0.434	0.666
Introjected regulation	4.21 (1.71)	4.39 (1.76)	*t* = 0.376	0.708
External regulation	3.00 (1.56)	2.70 (1.61)	*t* = -0.713	0.479
Attitude	5.95 (1.09)	6.02 (0.91)	*t* = 0.244	0.808
Intention	6.10 (0.82)	5.73 (1.45)	*t* = -1.066	0.291
PBC	5.57 (1.43)	5.57 (1.23)	*t* = 0.005	0.996
Subjective norms	4.50 (1.54)	3.86 (1.61)	*t* = -1.489	0.142
Effort	5.31 (1.28)	5.20 (1.41)	*t* = -0.302	0.763
Autonomy satisfaction	5.81 (0.88)	5.97 (1.13)	*t* = 0.580	0.564
Autonomy frustration	3.13 (1.14)	3.10 (1.55)	*t* = -0.081	0.936
Competence satisfaction	5.26 (1.19)	5.52 (1.45)	*t* = 0.700	0.487
Competence frustration	2.38 (1.17)	2.89 (1.66)	*t* = 1.230	0.224
Relatedness satisfaction	5.93 (0.83)	5.79 (1.04)	*t* = -0.514	0.610
Relatedness frustration	2.11 (1.57)	2.68 (1.49)	*t* = 1.384	0.172
Vigorous MET-min/week	2108.00 (2008.24)	2607.18 (2196.76)	*t* = 0.850	0.399
Moderate MET-min/week	719.00 (693.56)	1013.23 (1055.46)	*t* = 1.126	0.265
Walking MET-min/week	971.30 (1122.96)	758.59 (635.14)	*t* = -0.947	0.347
Total MET-min/week	3960.74 (2387.29)	4444.57 (2618.46)	*t* = 0.677	0.502

*Notes. PAS* Perceived autonomy support, *PCS* Perceived competence support, *PRS* Perceived relatedness support, *PBC* Perceived behavioral control, *MET* Metabolic equivalent task

#### Attrition check

Table 2 shows characteristics of the participants who remained in the study and those who were lost to follow-up. Results of the independent samples *t*-test revealed no significant baseline differences in most of the study variables (*t* = -1.385–1.729, *p* > 0.089). However, students who remained in the study reported significantly (*t* = -2.396–2.118, *p* > 0.047) higher perceived competence support from parents, lower autonomy frustration and lower leisure-time total PA at baseline than those who were lost in follow-up. Based on the chi-square test results, there was no significant difference in the proportion of male and female students between the students who remained in the study and those who were lost to follow-up (*χ*^*2*^ = 0.295, *p* = 0.587). The overall attrition rate in the study was 17.6 per cent and there was no significant difference in attrition between study groups (*χ*^*2*^ = 0.273, *p* = 0.601).

**Table 2:** Characteristics of the participants who remained in the study and those who were lost in follow-up

Variable	Remained in the study (*n* = 56), *M* (SD)	Lost in follow-up (*n* = 12), *M* (SD)	*t* or *χ*^*2*^ value	*p*
Age (in years)	12.56 (0.76)	12.25 (0.45)	*t* = 1.366	0.177
Gender (boy/girl)	23/32	4/8	*χ2* = 0.295	0.587
Group (control/intervention)	37/19	9/3	*χ2* = 0.273	0.601
PAS from parents	6.05 (0.90)	5.55 (1.11)	*t* = 1.538	0.130
PCS from parents	5.97 (0.88)	5.20 (1.68)	*t* = 2.118	0.039
PRS from parents	6.30 (0.92)	5.74 (1.10)	*t* = 1.698	0.095
Intrinsic motivation	5.57 (1.32)	4.73 (2.03)	*t* = 1.729	0.089
Identified regulation	5.70 (1.44)	5.68 (1.40)	*t* = 0.046	0.963
Introjected regulation	4.24 (1.77)	4.77 (1.54)	*t* = -0.932	0.355
External regulation	2.75 (1.57)	3.05 (1.72)	*t* = -0.566	0.574
Attitude	6.04 (0.97)	5.79 (0.97)	*t* = 0.780	0.439
Intention	5.88 (1.21)	5.73 (1.60)	*t* = 0.364	0.717
PBC	5.58 (1.29)	5.55 (1.35)	*t* = 0.076	0.939
Subjective norms	4.07 (1.64)	4.14 (1.50)	*t* = -0.123	0.902
Effort	5.24 (1.33)	5.20 (1.53)	*t* = 0.095	0.925
Autonomy satisfaction	6.02 (0.98)	5.48 (1.24)	*t* = 1.563	0.123
Autonomy frustration	2.92 (1.31)	4.00 (1.58)	*t* = -2.396	0.020
Competence satisfaction	5.35 (1.43)	5.82 (0.93)	*t* = -1.032	0.306
Competence frustration	2.63 (1.48)	3.09 (1.75)	*t* = -0.898	0.373
Relatedness satisfaction	5.92 (0.96)	5.48 (0.97)	*t* = 1.383	0.172
Relatedness frustration	2.34 (1.38)	3.05 (2.03)	*t* = -1.385	0.171
Vigorous MET-min/week	821.04 (859.73)	880.80 (729.83)	*t* = -1.314	0.194
Moderate MET-min/week	849.80 (875.89)	1225.60 (1274.46)	*t* = -1.140	0.259
Walking MET-min/week	2274.29 (2073.90)	3240.00 (2335.81)	*t* = -0.214	0.831
Total MET-min/week	3996.60 (2538.54)	5812.28 (1980.27)	*t* = -2.028	0.047

*Notes. PAS* Perceived autonomy support, *PCS* Perceived competence support, *PRS* Perceived relatedness support, *PBC* Perceived behavioral control, *MET* Metabolic equivalent task

### Main analysis

The results of repeated measures ANOVAs are presented in Table 3. Since the randomization check revealed a significant difference in the proportion of male and female students across the intervention and control groups, gender of participants was used as a covariate in each of the ANOVA analyses and was treated as another between-subject variable.

**Table 3:** Differences in study variables between the intervention and control group across three measuring occasions

Dependent variables	Baseline M (SD)	Post-intervention M (SD)	Follow-up M (SD)	F^a^	Partial η^2 (a)^	F^b^	Partial η^2 (b)^	F^c^	Partial η^2 (c)^
*PAS from parents*				1.148	0.027	1.203	0.028	0.428	0.010
Control group (*n* = 27)	6.04 (0.97)	5.97 (1.00)	5.87 (1.28)						
Intervention group (*n* = 17)	6.04 (0.88)	6.19 (0.87)	6.19 (0.63)						
*PCS from parents*				0.876	0.021	0.026	0.000	0.421	0.010
Control group (*n* = 27)	5.94 (1.03)	5.82 (1.15)	5.78 (1.19)						
Intervention group (*n* = 16)	5.95 (0.75)	6.17 (0.76)	6.09 (0.87)						
*PRS from parents*				0.521	0.012	0.732	0.017	0.119	0.003
Control group (*n* = 29)	6.23 (1.08)	6.30 (0.97)	6.09 (1.14)						
Intervention group (*n* = 17)	6.42 (0.68)	6.25 (0.93)	6.28 (0.68)						
*Intrinsic motivation*				3.095*	0.069	1.003	0.023	0.078	0.002
Control group (*n* = 29)	5.52 (1.36)	5.64 (1.28)	5.53 (1.30)						
Intervention group (*n* = 16)	5.63 (1.43)	5.25 (1.58)	5.63 (1.34)						
*Identified regulation*				0.469	0.011	1.589	0.037	0.074	0.002
Control group (*n* = 27)	5.69 (1.43)	5.43 (1.59)	5.70 (1.54)						
Intervention group (*n* = 17)	5.91 (1.39)	5.71 (1.39)	5.82 (1.20)						
*Introjected regulation*				3.107*	0.066	0.317	0.007	0.405	0.009
Control group (*n *= 30)	4.23 (1.89)	4.45 (1.71)	4.70 (1.56)						
Intervention group (*n* = 17)	4.29 (1.69)	3.94 (1.70)	3.62 (1.69)						
*External regulation*				0.511	0.011	0.010	0.000	0.112	0.003
Control group (*n* = 30)	2.58 (1.55)	2.52 (1.40)	2.67 (1.67)						
Intervention group (*n* = 17)	3.03 (1.66)	2.68 (1.46)	2.76 (1.38)						
*Attitude*				1.320	0.030	0.868	0.020	0.046	0.001
Control group (*n* = 28)	6.06 (0.86)	5.89 (0.96)	6.11 (0.88)						
Intervention group (*n* = 17)	5.96 (1.16)	6.00 (0.94)	5.98 (0.96)						
*Intention*				4.838**	0.103	2.497	0.056	1.397	0.032
Control group (*n* = 28)	5.66 (1.47)	5.29 (1.58)	5.82 (1.35)						
Intervention group (*n* = 17)	6.15 (0.70)	5.88 (1.02)	5.56 (1.47)						
*PBC*				0.702	0.016	0.158	0.004	0.066	0.002
Control group (*n* = 28)	5.61 (1.25)	5.50 (1.20)	5.48 (1.06)						
Intervention group (*n* = 17)	5.56 (1.52)	5.91 (0.87)	5.41 (1.61)						
*Subjective norms*				0.492	0.012	0.916	0.022	2.173	0.052
Control group (*n* = 27)	3.72 (1.69)	3.39 (1.56)	3.59 (1.48)						
Intervention group (*n* = 16)	4.71 (1.65)	4.41 (1.57)	4.16 (1.47)						
*Effort*				3.473**	0.080	2.709	0.063	0.022	0.000
Control group (*n* = 26)	5.08 (1.39)	4.60 (1.36)	4.90 (1.18)						
Intervention group (*n* = 17)	5.29 (1.34)	4.65 (1.51)	4.65 (1.66)						
*Autonomy satisfaction*				0.874	0.021	0.250	0.006	0.165	0.004
Control group (*n* = 28)	6.17 (1.00)	6.02 (1.17)	5.61 (1.43)						
Intervention group (*n* = 16)	5.81 (0.78)	5.81 (0.83)	5.69 (1.05)						
*Autonomy frustration*				2.987*	0.066	0.095	0.002	0.021	0.000
Control group (*n* = 29)	2.78 (1.25)	2.50 (1.12)	3.16 (1.54)						
Intervention group (*n* = 16)	3.00 (1.19)	2.83 (1.05)	2.61 (0.99)						
*Competence satisfaction*				0.204	0.005	1.921	0.045	0.222	0.005
Control group (*n* = 29)	5.34 (1.59)	5.59 (1.04)	5.64 (1.29)						
Intervention group (*n* = 15)	5.28 (1.32)	5.52 (1.33)	5.60 (1.28)						
*Competence frustration*				0.267	0.006	1.296	0.031	0.062	0.002
Control group (*n* = 29)	2.83 (1.66)	2.67 (1.45)	2.84 (1.59)						
Intervention group (*n* = 15)	2.37 (1.18)	2.37 (1.31)	2.50 (1.65)						
*Relatedness satisfaction*				0.989	0.023	1.580	0.036	0.034	0.000
Control group (*n* = 29)	5.91 (1.05)	5.76 (1.15)	5.73 (1.27)						
Intervention group (*n* = 16)	6.06 (0.82)	5.88 (1.15)	5.80 (1.33)						
*Relatedness frustration*				0.982	0.025	0.015	0.000	2.777	0.066
Control group (*n* = 25)	2.54 (1.26)	2.66 (1.32)	2.77 (1.55)						
Intervention group (*n* = 17)	2.06 (1.48)	1.84 (1.03)	1.68 (0.75)						
*Vigorous MET-min/week*				1.849	0.050	0.782	0.022	0.012	0.000
Control group (*n* = 23)	2815.65 (2311.48)	2102.26 (1698.60)	2307.48 (1922.94)						
Intervention group (*n* = 15)	1754.67 (1925.86)	2644.80 (4016.89)	3222.40 (3654.31)						
*Moderate MET-min/week*				0.248	0.007	0.502	0.015	0.967	0.028
Control group (*n* = 22)	1165.45 (1010.22)	1022.73 (1207.16)	1013.64 (909.64)						
Intervention group (*n* = 15)	782.67 (779.41)	682.13 (418.15)	749.87 (750.89)						
*Walking MET-min/week*				0.461	0.012	1.173	0.031	0.005	0.000
Control group (*n* = 25)	883.74 (706.17)	1031.98 (970.44)	1091.51 (901.52)						
Intervention group (*n* = 15)	873.40 (1226.05)	623.48 (1015.67)	925.76 (972.93)						
*Total MET-min/week*				1.478	0.047	0.362	0.012	0.208	0.007
Control group (n = 19)	5264.68 (2865.69)	4415.39 (2880.21)	4482.29 (2810.09)						
Intervention group (*n* = 14)	3518.71 (2337.06)	4034.09 (4377.02)	5208.24 (4385.91)						

*Notes. PAS* Perceived autonomy support, *PCS* Perceived competence support, *PRS* Perceived relatedness support, *PBC* Perceived behavioral control, *MET* Metabolic equivalent task, *M* Mean value, *SD* Standard deviation, *partial η2* Partial eta squared, a measure of effect size

^a^Refers to time x study group interaction effect

^b^Refers to time main effect

^c^Refers to study group main effect

^*^p = 0.05; **p < 0.05

Results of the repeated measures ANOVAs showed a significant, albeit borderline, time-by-study group interaction effect on intrinsic motivation (*F*(2, 84) = 3.095, *p* = 0.050), introjected regulation (*F*(2, 88) = 3.107, *p* = 0.050) and autonomy frustration (*F*(2, 84) = 2.987, *p* = 0.056). Subsequent post hoc pairwise comparisons using the Bonferroni correction, however, revealed no significant differences between children in the intervention group and the control group at any of the follow-up time points. Regarding intrinsic motivation, post-intervention mean value is higher for the control group. However, for the mean values of introjected regulation and autonomy frustration the trend indicates a decline in the intervention group, matching our assumptions.

Contrary to expectations, there were statistically significant time-by-study group interaction effects on intention (*F*(2, 84) = 4.838, *p* = 0.010) and effort (*F*(2, 80) = 3.473, *p* = 0.036) towards leisure-time PA. In terms of intention, subsequent post hoc tests using Bonferroni correction revealed no significant differences between children in the intervention group and the control group at any of the follow-up time points. For the mean values of intention towards leisure-time PA, the trend indicates a decline in the intervention group. With effort, time had a statistically significant effect on the outcome comparing baseline and post-intervention measuring occasions (*t* = 3.026, *p* = 0.010), with the results of post hoc comparisons averaged over group and gender. Also, in post hoc pairwise comparisons of time-by-study group interaction effects (results averaged over gender), significant differences were found comparing baseline vs post-intervention (*t* = 3.109, *p* = 0.039) and baseline vs follow-up (*t* = 3.123, *p* = 0.037) measuring occasions for the intervention group. The mean values for effort towards leisure-time PA in the intervention group were higher at baseline than at post-intervention and one-month follow-up, indicating significant decrease.

Regarding the main behavioural outcome measure physical activity, repeated measures ANOVAs did not reveal neither statistically significant main effects for time or study group nor statistically significant time-by-study group interaction effect. However, the students in the intervention group did demonstrate a positive, although not statistically significant trend in weekly vigorous physical activity at post-intervention and one-month follow-up.

## Discussion

Parental support is significantly correlated to children’s PA levels [26, 27]. Drawing from SDT, when a child’s basic psychological needs in PA are fulfilled, it facilitates the development of intrinsic motivation towards PA [17]. Hence, educating parents about need-supportive behaviours should help increase children’s PA. In this study we tested an online need-supportive training for parents expecting a positive effect on children’s leisure-time PA. A positive, albeit statistically insignificant trend can be seen in vigorous and total MET-minutes per week in the intervention group (Table 3), so it seems the need-supportive parenting program has some positive influence on the PA of children. However, we were not able to demonstrate statistically significant intervention effect on students’ MVPA, similarly to “Active 1 + FUN” and FRESH studies that also aimed to increase children’s PA through a family-based intervention [29, 31]. Like our intervention, the FRESH study was carried out online [31].

According to SDT, satisfying the basic psychological needs enables the development of more autonomous forms of motivation [17]. As a result of the parents’ training, we expected positive changes in intrinsic motivation and lower perceptions of controlled forms of motivation in children. Consistent with our expectations, students in the intervention group scored lower in introjected regulation, an extrinsic form of motivation, compared to students in the control group. However, no differences were found in external motivation, which is an even less self-determined form of motivation. In introjected regulation, behaviour is regulated by internal pressures to feel pride or avoid guilt or shame [15]. In the context of PA this could mean going for a walk because otherwise one would feel shame for being lazy or participating in training to win a contest. Compared to introjected regulation, intrinsic motivation is more likely to be maintained over time and thus should be aimed for when attempting to increase children’s PA.

Previous research has shown that need-supportive behaviours from PE teachers and peers enhance need satisfaction and lower need frustration for students in the PE context [48, 51]. We hypothesized that also children whose parents attended the need-supportive parenting training would score higher on need satisfaction and lower on need frustration compared to children in the control group. According to expectations students in the control group showed higher autonomy frustration at post-intervention and follow-up. This means that the children in the control group experienced more external pressure towards leisure-time PA and less perceived possibilities to choose the activities according to their own interests (e.g., “I feel I have to do a lot of things I don’t want to”). Therefore, it seems that parents using specific techniques to support children’s basic psychological needs for autonomy, competence, and relatedness, help relieve external pressure and support the development of more autonomous forms of motivation in students. To explicitly mention some techniques, the use of non-controlling language, providing choice and helping the child identify sources of external pressure allow the child to feel more in control, thus reducing autonomy frustration and controlled forms of motivation.

Regarding perceptions of need-supportive behaviour from parents, attitude and perceived behavioural control, no statistically relevant changes emerged. These findings are unexpected as previous research by Tilga and colleagues has demonstrated that a web-based autonomy-supportive training for teachers enhanced students’ perceptions of teachers’ autonomy-supportive behaviours [35]. As opposed to quite structured PE classes, domestic everyday life may not offer appropriate possibilities to try out new communication techniques discussing PA so regularly. In addition, it may take more time for the children to adjust to the new communication style by parents and change their perceptions accordingly. In further research, we aim to add another data collection point 5 months after the intervention to capture the possible long-term effects on children’s PA and psychological measures. Schneider and colleagues [61] conducted a study in the school setting, training PE teachers to use autonomy-supportive techniques. To point out the differences, the mean age of participating students was a bit higher than in our study (14.5 years vs 12.5 years), the need-supportive intervention was delivered by PE teachers vs parents in our program and the intervention was delivered face-to-face as opposed to our online approach. However, similarly to our study, Schneider and colleagues aimed to promote leisure-time PA of students by providing need support from social agents. They expected positive change in intrinsic motivation, other constructs from the trans-contextual model of motivation, and leisure-time PA, but were not able to obtain expected results. Schneider and colleagues argued that one possible reason for the null result was the baseline level of perceived autonomy support already being very high, leaving little room for improvement [61]. Similarly, the baseline values for perceptions of need-supportive behaviours from parents, attitude and perceived behavioural control in our study were also extremely high (on a 7-point scale > 5.90 for perceived need-support and attitude, > 5.50 for perceived behavioural control), being one possible explanation for the lack of significant positive changes. Both Schneider and colleagues’ and our study imply that changing student perceptions of need-supportive behaviour from social agents takes time, and the larger effect may come from reducing controlling behaviour (e.g., in our case the decreased autonomy frustration is the intervention group).

Inconsistent with our expectations, we found that intention and effort towards out-of-school PA were higher in the control group in the follow-up measuring occasion. One possible explanation could be seasonality regarding outdoor PA as both intention and effort are associated with actual PA. There are substantial seasonal differences in Estonia with rainy autumns, often snowy and windy winters, and moderately warm summers. It is also worth mentioning that from November to February daylight is scarce. Research has shown winter to be associated with lower levels of PA, especially among girls [62]. In our study, we had the first measuring occasion in October, the second in December, and the third in January. However, there were proportionally more boys in the intervention group than in the control group and as the boys’ PA levels should be more stable throughout the year, it is difficult to establish the effect of seasonality on our results.

The study has also some limitations. Firstly, we only used questionnaires for measurement, including collecting data about PA behaviour. The reason for using questionnaires solely is the aim to recruit participants as widely as possible. However, research has shown that in self-report measures participants frequently overestimate their PA levels as compared to accelerometer-measured data [63]. It seems self-report questionnaires to estimate PA levels could be used in older adolescents, especially in order to determine non-compliance with the recommended guidelines [64]. Objective measurement using accelerometers would add accuracy, at least partially to determine correlations between instruments. This is planned for future research.

The reasons for finding few intervention effects in post hoc tests can be attributed to the interaction effects being only borderline statistically significant, large standard deviations, the small number of participants in the study and uneven sample size in study groups. This does certainly reduce the statistical power of the study and the generalizability of the results. We found it difficult to get parents to participate in the training program, an obstacle also encountered by van Sluijs and colleagues [32]. In the pilot study, we used only school-based recruitment, approaching schools beforehand in written form and then visiting relevant classes in person. The results from our study make it clear that in further research it is necessary to employ several methods for recruitment. It is important to avoid relying only on children to communicate the aim, form, and content of the intervention to parents [30]. In further research we plan to include different channels for effective recruitment (information to children via schools, information to parents via schools’ online study systems and email lists, participating in parents meeting directly, posting in social media parenting and community groups, etc.) resulting in possible participants seeing the information on several occasions and being able to directly communicate with the research team.

Another limitation of the study is the significant difference in the proportion of male and female students between the intervention and control groups. We cluster-randomized by school, not knowing beforehand how many students voluntarily enroll and what the proportion of male and female students would be. This does impact the generalizability of the results.

A possibility for improvement is related to missing values. In the pilot study we used pen-and-paper questionnaires and had randomly missing answers to questions throughout questionnaires, reducing the reliability of the obtained findings. In further research we plan to use online questionnaires as this allows marking all answers as required. Tilga and colleagues have successfully used this approach on 11–15-year-old students previously [35].

Some limitations also apply to the used questionnaire. We adapted perceived competence and perceived relatedness support scales from Standage and colleagues [45] to refer specifically to parents. The reliability of the used scales was assessed using Cronbach’s alpha coefficient and the values were between 0.873 and 0.934, which suggests reliability of the scales used. Secondly we slightly modified the BPNSNF scale by Chen and colleagues [49] that has been adapted to the PE context by Haerens and colleagues [50] and we slightly modified the adapted version to refer specifically to leisure-time PA. The reliability of the used scales was assessed using Cronbach’s alpha coefficient and the values were between 0.856 and 0.924, which suggests reliability of the scales used. Future studies with larger sample size are needed to validate the adapted versions of the Perceived Need Support Scale and BPNSNF.

Further research should be carried out to test the intervention in other domains, where messages related to PA are delivered, such as PE in schools. To achieve the highest impact on students’ PA levels, interventions in several domains (e.g., home and school settings) could be combined. This would mean creating a need-supportive social environment in several contexts for the children, thus leading to a comprehensive approach to promoting their PA levels. Such supportive environments enable satisfying all three basic psychological needs, making it possible to reach optimal performance and wellbeing for the children [16]. Potential challenges of implementing the intervention in different settings include recruiting a sufficient number of participants and students not wearing the accelerometers for 7 consecutive days during the census period (e.g., forgetting to wear the device).

Future research could be based on a newer classification of motivational behaviours by Ahmadi and colleagues set in the educational context [65]. The advantages of this classification include describing need thwarting behaviours in addition to need supporting techniques, more experts participating in expert panels, and higher criteria for reaching consensus. The inclusion of need-thwarting behaviours enables practitioners to identify which behaviours to avoid. Previous research has shown that controlling behaviour attenuates the effect of autonomy-support on students’ health-related outcomes [66]. This classification system gives clear definitions of each behaviour and provides estimates of how effective it might be for promoting motivation, thus enabling researchers and practitioners to select the most relevant techniques for the aim of the potential intervention.

The results of the study are expected to contribute to informing the development of future interventions and educational programs for parents to promote PA in children and adolescents. The online approach makes it possible to reach large audiences and enables self-paced learning for participating parents. It is vital to find effective and cost-effective ways to support PA, that can also be adapted to various countries and age groups.

## Conclusions

This study tested an online need-supportive training for parents to enhance children's leisure-time physical activity and autonomous motivation. There was a positive trend in vigorous and total MET-minutes per week for children in the intervention group, but no statistically significant intervention effect. However, the parents' training resulted in lower perceptions of introjected regulation and autonomy frustration in children, indicating the development of more autonomous forms of motivation towards leisure-time PA in children. These findings suggest that while the parent’s need-supportive training may not have a significant impact on physical activity, it can increase children's intrinsic motivation and reduce controlled forms of motivation. Future research should aim to address the limitations of the current study, including the use of objective measures of physical activity and recruiting larger sample sizes. To improve the generalizability of the findings, future research could test the intervention in other domains beyond leisure-time PA, such as school PE lessons.

### Supplementary Information

**Additional file 1.**

## Data Availability

The datasets generated during and analysed during the current study are available in the Open Science Framework (OSF) repository, 10.17605/OSF.IO/DG534[67].

## Abbreviations

IPAQ: International Physical Activity Questionnaire
MBCT: Motivation and behavioural change technique
MET: Metabolic Equivalent Task
MVPA: Moderate-to-vigorous physical activity
PA: Physical activity
PE: Physical education
SDT: Self-determination theory
TCM: Trans-contextual model of motivation
WHO: World Health Organization
