# Supplementary material for: Web-based need-supportive parenting program to promote physical activity in secondary school students: a randomized controlled pilot trial
Source: BMC Public Health. 2023 Aug 25;23:1627. doi: 10.1186/s12889-023-16528-4 (PMC10463639; doi:10.1186/s12889-023-16528-4)
Supplement: Supplementary file 1 — Additional file 1. [file 12889_2023_16528_MOESM1_ESM.docx]

**Additional file 1**

Description of the web-based need-supportive parenting program

The program was carried out in the learning platform Moodle, and it lasted for 6 weeks. Every week we published 3 educational videos. At the end of each week, there was a 3-question quiz on that week's videos to test the parents’ understanding of the techniques. In addition to the test, a weekly task was to briefly write in the forum whether the parents tried to apply the presented techniques and how they succeeded.

After completing the study program, we expected parents:

- to understand how the satisfaction of a child's basic psychological needs and intrinsic motivation towards leisure-time physical activity (PA) are related;
- to understand the importance of a child's intrinsic motivation from the point of view of leisure-time PA;
- to have familiarized themselves with various techniques for supporting the child's basic psychological needs in the context of leisure-time PA, and to know how to apply them.

**Table.** The content of educational videos that were based on Motivation and Behavior Change Techniques (MBCT) described by Teixeira and colleagues (2020), and adapted to the context of parent-child interaction and leisure-time PA.

| Name of technique (from Teixeira *et al.* 2020) | Expected benefit | Description of educational video | Length (min:sec) |
| --- | --- | --- | --- |
|  |  |  |  |
| *Autonomy-support techniques* |  |  |  |
| 1. Elicit perspectives on condition or behavior | The child gains self-knowledge and the parent is able to more effectively support him/her in future. | Parent encourages the child to think about his/her current leisure-time PA behavior and motivating factors behind it. | 01:52 |
| 2. Prompt identification of sources of pressure for behavior change | The child learns to identify whether the initiative is intrinsic or external/introjected. The parent can change his/her behavior if necessary. | Parent helps the child to identify any sources of pressure (including parents themselves, peers, teachers etc.) regarding leisure-time PA behavior. | 02:40 |
| 3. Use noncontrolling, informational language | The parent avoids being a source of pressure, the child feels more in control. | Parent interacts with the child about leisure-time PA in two different ways: (1) using controlling language and pressure; (2) using noncontrolling language. | 02:13 |
| 4+5. Explore life aspirations and values/ Provide a meaningful rationale | The child brings forward motives that could lead to the formation of autonomous motivation. The parent understands the child's long-term interests better. | Parent discusses with the child his/her goals in different areas of life and helps connect the goals with being physically active. | 02:48 |
| 6. Provide choice | The child decides how to move forward and takes responsibility for being more active in his/her leisure time. | Parent discusses with the child the possibilities s/he has for being physically more active. The child decides which options to explore and whether to make the change. | 01:56 |
| 7. Encourage the person to experiment and self-initiate the behavior | The child will more likely find a form of movement s/he enjoys, and this increases autonomous motivation to be more active. | Parent encourages the child to experiment with different ways of movement to facilitate him/her finding a motivating leisure-time PA activity. | 02:02 |
|  |  |  |  |
| *Relatedness-support techniques* |  |  |  |
| 8. Acknowledge and respect perspectives and feelings | The parent shows attention and respect for the child's attitudes, thoughts, and feelings, creating a trusting and warm environment. | Parent interacts with the child about what is difficult for him/her regarding leisure-time PA and what s/he enjoys. | 02:19 |
| 9+13. Encourage asking of questions/ Providing opportunities for ongoing support | The parent demonstrates care and presence. This promotes trust and enables developing an open and cooperative relationship. | Parent assures the child that s/he is always there to support him/her regarding leisure-time PA when s/he needs it, and encourages him/her to ask questions about leisure-time PA. | 02:10 |
| 10. Show unconditional regard | The parent demonstrates care and warm relations in the family are maintained. | Parent expresses understanding and support to the child although s/he had not met his/her goals regarding leisure-time PA. | 01:34 |
| 11. Demonstrate/show interest in the person | The child feels that his/her experiences are important to the parent and that the parent values him/her as a person. | Parent expresses pleasure at interacting with the child and asks for his/her opinion about different life events. | 01:30 |
| 12. Use empathic listening | The parent promotes trust, the development of an open and cooperative relationship and shows respect for the child. | Parent reflects the child's feelings when s/he is upset, and they work together to find a solution to the problem. | 02:15 |
| 14. Prompt identification and seek available social support | The parent helps the child find necessary self-confidence to overcome possible difficulties and achieve his/her goals. | Parent discusses with the child the people most influencing his/her leisure-time PA, and who could help to increase his/her PA levels and how. | 03:02 |
|  |  |  |  |
| *Competence-support techniques* |  |  |  |
| 15. Address obstacles for change | The child's self-confidence increases, and when a problem arises, s/he is more ready to deal with it. | Parent discusses with the child which obstacles prevent him/her from being physically more active in his/her leisure-time, and how to overcome these difficulties. | 02:32 |
| 16. Clarify expectations | Comprehensibly formulated expectations provide the child structure and reduce the likelihood of failure, s/he feels more competent. | Parent helps the child to clearly define his/her own expectations regarding leisure-time PA | 02:04 |
| 17+19. Assist in setting optimal challenge/ Help develop a clear and concrete plan of action | Optimal goals and a plan of action provide the child structure and reduce the likelihood of failure, s/he feels more competent. | Parent discusses with the child which meaningful and attainable goals s/he has regarding leisure-time PA and helps him/her develop a plan of action to achieve the goals. | 02:50 |
| 18. Offer constructive, clear, and relevant feedback | Appropriate and positive feedback offers the child encouragement and information to manage his/her behavior in the future. | Parent provides the child with relevant and non-judgmental feedback about his/her goals and performance regarding leisure-time PA. | 02:18 |
| 20. Promote self-monitoring | Self-monitoring offers the child confirmation that s/he is moving in the right direction, it gives him/her a sense of success and reinforces his/her self-awareness. | Parent and child talk about how the child could monitor his/her own progress regarding leisure-time PA. | 02:23 |
| 21. Explore ways of dealing with pressure | The child learns to regulate the effects of external pressure better, his/her self-confidence and belief in his/her own competence increases. | Parent helps the child identify sources of external pressure regarding leisure-time PA and they discuss how the child himself/herself could make PA more motivating for him/her. | 02:30 |
|  |  |  |  |
|  |  | Average length of videos | **02:16** |
